# CHAMP1 disorder is associated with a complex neurobehavioral phenotype including autism, ADHD, repetitive behaviors and sensory symptoms

**DOI:** 10.1093/hmg/ddac018

**Published:** 2022-03-11

**Authors:** Tess Levy, Bonnie Lerman, Danielle Halpern, Yitzchak Frank, Christina Layton, Jessica Zweifach, Paige M Siper, Joseph D Buxbaum, Alexander Kolevzon

**Affiliations:** Seaver Autism Center for Research and Treatment, Icahn School of Medicine at Mount Sinai, New York, NY 10029, USA; Department of Psychiatry, Icahn School of Medicine at Mount Sinai, New York, NY 10029, USA; Seaver Autism Center for Research and Treatment, Icahn School of Medicine at Mount Sinai, New York, NY 10029, USA; Department of Psychiatry, Icahn School of Medicine at Mount Sinai, New York, NY 10029, USA; Seaver Autism Center for Research and Treatment, Icahn School of Medicine at Mount Sinai, New York, NY 10029, USA; Department of Psychiatry, Icahn School of Medicine at Mount Sinai, New York, NY 10029, USA; Seaver Autism Center for Research and Treatment, Icahn School of Medicine at Mount Sinai, New York, NY 10029, USA; Department of Psychiatry, Icahn School of Medicine at Mount Sinai, New York, NY 10029, USA; Seaver Autism Center for Research and Treatment, Icahn School of Medicine at Mount Sinai, New York, NY 10029, USA; Department of Psychiatry, Icahn School of Medicine at Mount Sinai, New York, NY 10029, USA; Seaver Autism Center for Research and Treatment, Icahn School of Medicine at Mount Sinai, New York, NY 10029, USA; Department of Psychiatry, Icahn School of Medicine at Mount Sinai, New York, NY 10029, USA; The Mindich Child Health and Development Institute, Icahn School of Medicine at Mount Sinai, New York, NY 10029, USA; Seaver Autism Center for Research and Treatment, Icahn School of Medicine at Mount Sinai, New York, NY 10029, USA; Department of Psychiatry, Icahn School of Medicine at Mount Sinai, New York, NY 10029, USA; The Mindich Child Health and Development Institute, Icahn School of Medicine at Mount Sinai, New York, NY 10029, USA; Seaver Autism Center for Research and Treatment, Icahn School of Medicine at Mount Sinai, New York, NY 10029, USA; Department of Psychiatry, Icahn School of Medicine at Mount Sinai, New York, NY 10029, USA; The Mindich Child Health and Development Institute, Icahn School of Medicine at Mount Sinai, New York, NY 10029, USA; Department of Genetics and Genomic Sciences, Icahn School of Medicine at Mount Sinai, New York, NY 10029, USA; Department of Neuroscience, Icahn School of Medicine at Mount Sinai, New York, NY 10029, USA; Seaver Autism Center for Research and Treatment, Icahn School of Medicine at Mount Sinai, New York, NY 10029, USA; Department of Psychiatry, Icahn School of Medicine at Mount Sinai, New York, NY 10029, USA; The Mindich Child Health and Development Institute, Icahn School of Medicine at Mount Sinai, New York, NY 10029, USA; Department of Pediatrics, Icahn School of Medicine at Mount Sinai, New York, NY 10029, USA

## Abstract

CHAMP1-related neurodevelopmental disorder, or CHAMP1 disorder, is a recently described genetic syndrome associated with developmental delay, intellectual disability, behavioral symptoms, medical comorbidities, and dysmorphic features. To date, literature has focused on medical review and dysmorphology but has yet to prospectively assess neurobehavioral core domains such as autism, or behavioral, language, cognitive, and sensory features. Here, we present deep phenotyping results for 11 individuals with CHAMP1 disorder, based on approximately 12 hours of remote clinician-administered assessments and standardized caregiver questionnaires. Diagnoses of autism spectrum disorder were given to 33% of participants; repetitive behaviors and sensory-seeking symptoms were prominent in this cohort. In addition, 60% of participants met the criteria for attention-deficit/hyperactivity disorder (ADHD). High rates of ADHD and relatively low rates of treatment suggest potential areas for intervention. This study represents the first prospective phenotyping analysis of individuals with CHAMP1 disorder. The utility of specific measures as clinical endpoints, as well as benefits and limitations of remote phenotyping, are described.

## Introduction

The rise of large-scale genomic studies and clinical genetic testing has led to an increase in the identification of specific genes that cause neurodevelopmental disorders (NDDs) (1,2). The diagnostic yield for genetic testing using chromosomal microarray and exome sequencing in individuals with intellectual disability (ID) and/or developmental delays (DD) is over 50% and around 30% for individuals with autism spectrum disorder (ASD) (3–9). Many of the genes associated with these NDDs converge on common processes, including synaptogenesis (e.g. *NLGN3*, *SHANK3*), chromatin remodeling (e.g. *CHD8*, *PAX6*), transcriptional regulation (*SMARCA4*, *FOXP1*) (10–17) and chromosome alignment and/or spindle assembly (e.g. *POGZ*, *KIF2A*). *CHAMP1*, a gene involved in proper chromosome segregation, has recently been associated with a neurodevelopmental disorder (18,19).

*CHAMP1* pathogenic variants were first identified in 2 of 1133 children with severe DD in the Deciphering Developmental Disorders study (20). The first study to specifically describe the CHAMP1 disorder phenotype was published in 2015 by Hempel *et al*. and included five individuals diagnosed with what was formerly named autosomal dominant mental retardation type 40 (MIM: 616579) (19). All exhibited ID, delayed speech development, dysmorphic features, hypotonia, and friendly behavior. Stereotyped behavior, decreased pain sensation, and microcephaly were also described. Another case series of five individuals was published in 2016 by Tanaka *et al*., which replicated the earlier findings and also described common features of hearing loss, behavioral abnormalities (e.g. hyperactivity, impulsivity and aggression), and sleep disturbance (21). In 2016, Isidor *et al*. reported an additional six individuals with a similar phenotypic presentation (22). The largest cohort to date was published by Garrity *et al*. in 2021 and reviewed the medical and dysmorphic features in 14 individuals with CHAMP1 disorder (23), adding features such as gastrointestinal abnormalities to the phenotype and highlighting a potential association between *CHAMP1* and cancer, based on one individual with leukemia.

*CHAMP1* remains a rare cause of NDDs, possibly accounting for 0.03% of NDD cases. Clinical laboratories have seen positive *CHAMP1* findings for 22 of 6,2 586 and 5 of 1,1 000 exomes at GeneDX and Ambry Genetics, respectively (24,25). However, *CHAMP1* is not currently present on many clinical ASD and/or NDD panels, presumably because it was only recently discovered and lacks strong phenotypic evidence for particular features such as ASD and epilepsy. Therefore, the syndrome is likely underdiagnosed.

While the majority of publications to date describe the developmental and medical phenotype of individuals with CHAMP1 disorder, the syndrome has not yet been prospectively characterized in detail. Additionally, while some literature describes ASD features, no studies prospectively diagnosed ASD or characterized ASD traits. Here, we present deep phenotyping results in 11 participants with CHAMP1 disorder, 8 novel to the literature and 3 previously published, with special interest in ASD symptomatology, additional behavioral findings, development, and regression. We also present a fully remote battery of assessments developed during the COVID-19 pandemic and detail the benefits and limitations of telehealth phenotyping. This is the first known cohort of its kind to be comprehensively characterized remotely.

## Results

**Figure 1:**
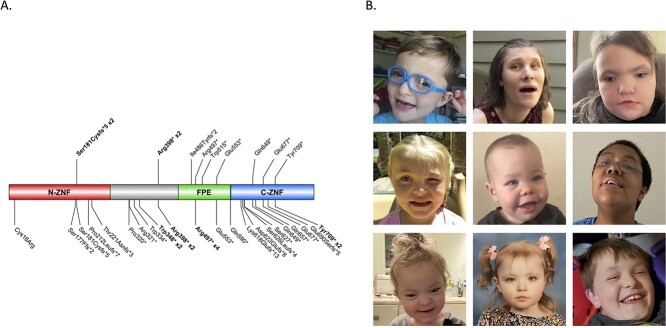
Genetic landscape and participants. (**A**) Individuals’ *CHAMP1* pathogenic variants mapped onto the *CHAMP1* gene. Variants above the gene are individuals in our cohort; variants below the gene are individuals reported in the literature. Bolded variants indicate recurrence in our cohort or the literature. (**B**) Photos of participants in our cohort.

All participants had pathogenic protein-truncating variants in *CHAMP1*, including eight nonsense and three frameshift variants (Fig. 1, Supplementary Material, Table S1). There were two recurrent variants in our cohort: two individuals with p.Ser181Cysfs^*^5, which was also reported in a different patient in the literature, and two individuals with p.Ile486Tyrfs^*^2, which has not been previously reported in the literature.

### Development and regression

**Figure 2:**
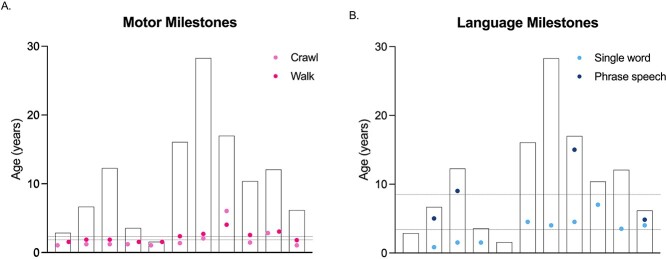
Motor and language milestones in the cohort. In each graph, bars represent the age of participant at the time of evaluation, dots represent the age of achievement of each skill, and dotted lines represent the average age of achievement for the group. (**A**) Light pink dots represent the age at crawling, dark pink dots represent the age of walking; the lower dotted line represents the average age of crawling, and higher dotted line represents the average age of walking. (**B**) Light blue dots represent the age at the first single word, dark blue dots represent the age of phrase speech achievement; the lower dotted line represents the average age of the first single word and higher dotted line represents the average age of phrase speech.

Motor milestones were delayed in all participants. Participants began to crawl between 12 and 72 months (21.9 ± 17.9) and began to walk between 18 and 48 months (26.6 ± 9.4) (Fig. 2, Supplementary Material, Table S2). All participants except one learned to crawl before walking. These two skills were achieved by all participants.

Nine of 11 participants had a minimum of single-word speech at the time of evaluation. Language milestones were delayed in all participants. First word was achieved between 18 and 84 months (41.8 ± 23.2). Only four participants had phrase speech, which developed between 58 and 180 months (101.5 ± 57.21) (Fig. 2). Participants without a single word were 27.2 ± 11.0 months old on average, and participants without phrase speech were 128.7 ± 113.3 months old on average. Daytime and nighttime bladder control was not achieved by any participants in our cohort. Bowel control was achieved by one participant at 11 years.

Overall, there was minimal evidence of regression in this cohort. Minor reported regressions included two individuals who lost babbling, one at 10 months and the other at 14 months; participants regained this skill at 16 and 24 months, respectively. Additionally, there was one participant who lost the ability to roll over at 11 months; this skill was regained at 22 months. Notably, there was no reported regression in social skills (e.g. social smile, eye contact), motor skills other than rolling over (e.g. sit without support, walk), or language skills other than babbling (e.g. use single words, use phrases).

### Cognitive and adaptive functioning

**Figure 3:**
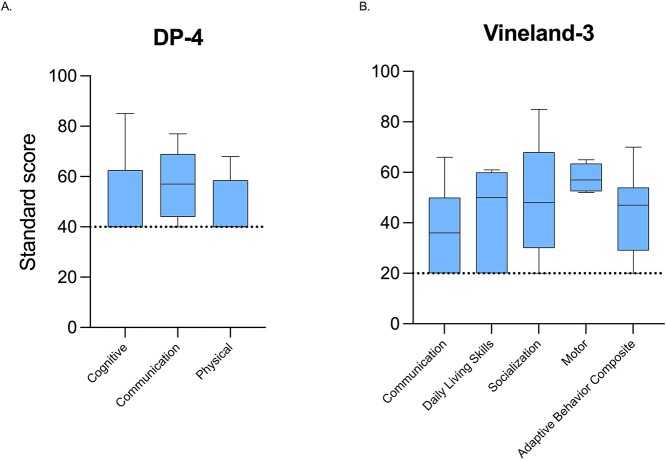
Cognitive and adaptive functioning results. Standard scores on the Developmental Profile-4 (**A**) and the Vineland-3 (**B**) are displayed. Dotted lines represent the floor of each assessment.

The DP-4 Cognitive domain standard scores ranged from 40 (floor) to 85 (49.8 ± 15.8) (Fig. 3). The youngest participant had the highest score.

Adaptive functioning was significantly affected in all participants. Scores on the Vineland-3 Adaptive Behavior Composite ranged from 20 (floor) to 70 (44.0 ± 17.0), where the youngest participant had the highest score. Comparing each domain, scores were the highest in the Socialization domain, which ranged from 20 to 85 (48.0 ± 21.6) and lowest in the Communication domain, which ranged from 20 to 66 (37.7 ± 16.0). Scores in the Daily Living Skills domain ranged from 20 to 61 (44.0 ± 16.6). The Motor domain was completed in individuals of age 7 years and younger (*n* = 5), with scores ranging from 52 to 65 (57.8 ± 5.6). Subdomain scores are shown in Table 1.

**Table 1:** Vineland-3 domain and subdomain scores

Subdomain	Range	Mean (SD)
Adaptive behavior composite	20–70	44.0 (17.0)
Communication domain		
Communication	20–66	37.3 (16.0)
Receptive	1–10	5.91 (4.1)
Expressive	1–7	1.82 (1.8)
Written (*n* = 9)	1–10	3.11 (3.4)
Daily living skills domain		
Daily living skills	20–61	44.0 (16.6)
Personal	1–7	2.09 (2.4)
Domestic (*n* = 9)	1–9	5.56 (2.6)
Community (*n* = 9)	1–11	3.78 (3.5)
Socialization domain		
Socialization	20–85	48.0 (22.6)
Interpersonal relationships	1–14	5.45 (4.4)
Play and leisure	1–13	3.82 (4.3)
Coping skills	1–12	6.80 (3.2)
Motor skills domain		
Motor	52–65	57.8 (5.6)
Gross motor (*n* = 5)	1–8	6.40 (1.5)
Fine motor (*n* = 5)	1–11	7.20 (2.3)
Maladaptive behavior domain		
Internalizing	19–22	20.44 (1.2)
Externalizing	18–22	20.00 (1.2)

Written, Domestic, and Community domains only administered to individuals over 3 years. Motor domain only administered to individuals under 7 years. Vineland-3 domains are measured in standard scores which have a population mean of 100 and standard deviation of 15; subdomains are measured in V-Scale scores which have a population mean of 15 and a standard deviation of 3. The first row in each domain is the standard score, followed by the subdomain V-scale scores.

### ASD symptomatology

**Figure 4:**
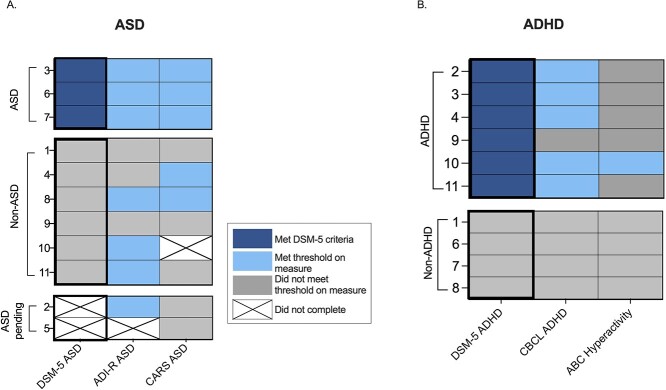
ASD and ADHD assessments. (**A**) Numbers on the left-hand side represent patient ID numbers. The first column represents Diagnostic and Statistical Manual for Mental Disorders, Fifth Edition (DSM-5) diagnosis of autism spectrum disorder (ASD), where dark blue color indicates a diagnosis of ASD, gray indicates no diagnosis of ASD, and crosses indicate that diagnosis was deferred. The second column represents the results from the Autism Diagnostic Interview-Revised (ADI-R), where light blue color indicates the participant surpassed the threshold for ASD on this assessment, gray indicates they did not surpass the threshold, and a cross indicates the measure was not completed. The third column represents results from the Childhood Autism Rating Scale, where the light blue indicates the participant surpassed the threshold for ASD on this assessment, gray indicates they did not surpass the threshold, and a cross indicates the measure was not completed. Abbreviations: ADI-R: Autism Diagnostic Interview-Revised; ASD: Autism Spectrum Disorder; CARS-2^obs^: Childhood Autism Rating Scale; DSM-5: Diagnostic and Statistical Manual of Mental Disorders, Fifth Edition. (**B**) Numbers on the left-hand side represent patient ID numbers. The first column represents DSM-5 diagnosis of ADHD where dark blue indicates a diagnosis of ADHD, and gray indicates no diagnosis of ADHD. The second column represents the results from the CBCL, where light blue color indicates the participant surpassed the threshold for the domain (*T* score > 65), and gray indicates they did not surpass the threshold. The third column represents results from the ABC, where the light blue indicates the participant surpassed the threshold for the domain (*T* score > 60), and gray indicates they did not surpass the threshold. *Abbreviations*: ABC: Aberrant Behavior Checklist; ADHD: Attention deficit hyperactive disorder; CBCL: Childhood Behavioral Checklist; DSM-5: Diagnostic and Statistical Manual of Mental Disorders, Fifth Edition; Vineland-3: Vineland Adaptive Behavior Scales, Third Edition.

Seven of 10 participants surpassed the cutoff on all the Autism Diagnostic Interview-Revised (ADI-R) domains. All 10 met the cutoff in the Abnormality of Development domain, 9 in Restricted and Repetitive Behavior (RRB), and 8 in Abnormalities in Reciprocal Social Interaction domain. In the Communication domain, four out of four met criteria on the Verbal algorithm and five out of six on the Nonverbal algorithm. On the Childhood Autism Rating Scale (CARS-2^obs^), three individuals met for severe ASD, two for mild–moderate ASD, and five did not surpass the threshold for ASD. On the psychiatric evaluation, 9 of 11 participants presented with at least one symptom of ASD. Overall, three participants met for a consensus Diagnostic and Statistical Manual for Mental Disorders, Fifth Edition (DSM-5) diagnosis of ASD. Specific DSM-5 criteria for each individual can be found in Supplementary Material, Table S3. Clinicians deferred one diagnosis until an in-person assessment is feasible, and one participant was deemed too young to determine. The ADI-R had 33% true positives, 33% false positives, and 33% true negatives; there were no false negatives (Fig. 4). The CARS-2^obs^ had 38% true positives, 25% false positives, and 38% true negatives; there were no false negatives when compared to consensus diagnosis.

On the Social Responsiveness Scale, Second Edition (SRS-2), 5 of 10 participants’ Total T-scores fell in the severe range, three in the moderate range and two in the normal range, with an average Total T-score of 73.5 ± 11.4. Five participants scored in the severe range in the Social Cognition domain (70.7 ± 13.6), four in the Social Motivation domain (67.1 ± 11.8), two in the Social Communication domain (68.6 ± 11.8), and one in the Social Awareness domain (61.1 ± 8.7). Eight of 10 participants scored in the severe range in the RRB domain; overall, the cohort had an average RRB T-score of 82.2 ± 13.4.

The total score on the Repetitive Behavior Scale-Revised (RBS-R) ranged from 2 to 71 (29.4 ± 19.8), with a total maximum score of 129. The cohort had the highest scores, proportionally, on the Ritualistic domain, scoring on average 6.0 ± 4.4 out of 18 total points. The next highest scores were within the Restricted Behavior and Sameness Behavior domains, where participants scored 3.6 ± 2.9 of 12 total points and 8.0 ± 6.3 of 33 total points, respectively. The cohort scored the lowest on the Stereotyped Behavior, Compulsive Behavior, and Self-Injury domains with 3.6 ± 2.4 of 18, 4.3 ± 4.0 of 24 and 3.7 ± 4.4 of 24 points.

The Sensory Assessment for Neurodevelopmental Disorders (SAND) Total Reported scores ranged from 6 to 33 (20.1 ± 9.3). Additionally, results indicated that participants had the most sensory seeking symptoms (7.8 ± 4.3), followed by hyporeactivity (6.3 ± 3.1) and hyperreactivity (6.0 ± 4.2) symptoms. In terms of sensory modalities, participants showed the most tactile symptoms (9.0 ± 2.5), followed by visual symptoms (5.8 ± 3.8) and auditory symptoms (5.3 ± 4.6). Compared to a typically developing cohort (*N* = 54, *M*_age_ = 5.4), individuals with CHAMP1 disorder had significantly more sensory features based on SAND interview scores in total and on every subdomain (*P* < 0.01). In the typically developing cohort, the average reported total score was 2.7 ± 2.3, reported hyperreactivity was 1.3 ± 1.5, hyporeactivity was 0.3 ± 0.7, and seeking was 1.1 ± 1.5. The average reported visual score in the typically developing cohort was 0.4 ± 0.9, reported tactile was 1.2 ± 1.8, and reported auditory was 1.1 ± 1.4.

Individual scores on the SP Total ranged from 328 to 536 (416.6 ± 63.0). In the Quadrant scores, all nine individuals had definite differences in Low Registration, seven in both Sensation Seeking and Sensory Sensitivity, and six in Sensation Avoiding. Caregivers reported the highest sensory behaviors on the SP in Touch Processing, where 9 of 9 individuals scored in the definite difference range. This was followed by Multisensory Processing, where eight individuals had definite differences; Vestibular Processing, where six individuals had definite differences; and Auditory and Visual Processing, where three individuals each had definite differences.

On the Sensory Experiences Questionnaire Version 3.0, participants scored highest, on average, in the Sensory Interests, Repetitions and Seeking Behavior domain, with an item mean of 2.5 (0.4) of 5. They scored higher in the Hyperresponsiveness domain (2.3 ± 0.6) than Hyporesponsiveness (2.2 ± 0.3), and they scored the lowest in the Enhanced Perception domain (2.0 ± 0.6). Participants demonstrated increased sensory symptoms in the Social Context (2.4 ± 0.4) compared to the Non-social Context (2.3 ± 0.4). Comparing sensory modalities, the cohort exhibited the highest scores in the Tactile domain (2.6 ± 0.3), followed by the Visual domain (2.3 ± 0.4), Vestibular/Proprioception domain (2.2 ± 0.4), Auditory domain (2.2 ± 0.6), and the Gustatory/Olfactory domain (2.2 ± 0.3).

### Language and communication

**Figure 5:**
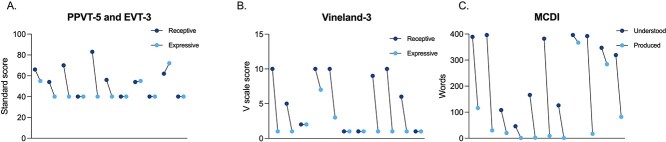
Receptive and expressive language abilities. Results from various language assessments. (**A**) Results from the PPVT-5 and EVT-3. Dark blue dots indicate participants’ standard scores on the PPVT-5, light blue dots represent participants’ scores on the EVT-3. Paired dots indicate one individual’s scores on both evaluations. The floor of the PPVT-5 and EVT-3 is 40. (**B**) Results from the Vineland-3 caregiver interview. Dark blue dots indicate participants’ Receptive Language subdomain scores, light blue dots indicate their Expressive Language subdomain scores. Paired dots represent scores for each participant. The floor of the Vineland-3 is 1. (**C**) Results from the MCDI. Dark blue dots represent words understood, light blue dots represent words produced. Paired dots represent scores for each participant on the MCDI. The floor of this assessment is 0. *Abbreviations*: EVT-3: Expressive Vocabulary Test Third Edition; PPVT-5: Peabody Picture Vocabulary Test Fifth Edition; MCDI: MacArthur Bates Communicative Indices; Vineland-3: Vineland Adaptive Behavior Scales, Third Edition.

Standard scores on the Peabody Picture Vocabulary Test, Fifth Edition (PPVT-5) ranged from 40 to 83 (56.6 ± 12.3), and 40 to 72 (46.2 ± 11.0) on the Expressive Vocabulary Test, Third Edition (EVT-3) (Fig. 5). Four individuals demonstrated higher receptive vocabulary skills (>12-point difference, per comparison report), and six had similar scores on both assessments.

Scores on the Vineland-3 Receptive Language subdomain ranged from 1 to 10 (5.9 ± 4.1), 1 to 7 on the Expressive Language subdomain (1.8 ± 1.8), and 1 to 10 on the Written Language subdomain (3.1 ± 3.4). On average, each participant’s Receptive Language score was more than one standard deviation (4.1 ± 3.6) greater than his or her Expressive Language score. Scores on the DP-4 Communication domain ranged from 40 to 77 (57.9 ± 12.7), with the youngest individual having the highest score (Fig. 3).

On the MacArthur–Bates Communicative Indices (MCDI), caregivers reported that participants understood 267.1 (138.9) words out of a total of 396 and produced 56.2 (88.8). On average, participants understood 191 more words than they could produce. While understanding more words than those produced is expected in early stages of development, participants tended to *disproportionately* understand a greater number of words than they could produce. Participants had an average of 13.6 (3.3) of 18 early actions and gestures (e.g. waves, nods head yes) and 23.6 (12.4) of 45 later actions and gestures (e.g. put telephone to ear, throw ball).

### Motor

The Beery Visual-Motor Integration, Sixth Edition (VMI-6) was completed by five participants, with standard scores ranging from 42 to 83 (50.8 ± 18.0); four of the five met the criteria for a visual motor integration disorder. The six individuals who could not complete the VMI-6 were not able to independently manipulate a pencil. On the DP-4 Physical domain, participants’ scores ranged from 40 to 68 (47.0 ± 11.1) (Fig. 3).

On the Developmental Coordination Disorder Questionnaire (DCDQ), participants on average scored 7.8 (2.0) on the Control During Movement domain (maximum 30), 4.1 (0.3) on the Fine Motor/Handwriting domain (maximum 20) and 8.9 (3.8) on the General Coordination domain (maximum 25). Overall, participants scored 20.8 (4.5) on the Total Score; all participants met the threshold for a developmental coordination disorder. On the Vineland-3 Motor domain, participants had standard scores ranging from 52 to 65 (57.8 ± 5.6). Participants showed similar fine and gross motor skills (Table 1); Gross Motor subdomain V scale scores ranged from 4 to 8 (6.4 ± 1.5) and Fine Motor from 5 to 11 (7.2 ± 2.3).

### Psychiatric and behavioral comorbidities

Six of 11 participants presented with inattention and hyperactivity and 5 of 11 with impulsivity. Consensus DSM-5 diagnoses of attention-deficit/hyperactivity disorder (ADHD) were given to 6 of 10 participants (Fig. 4, Supplementary Material, Table S4). One participant was too young for a diagnosis. Three participants had a history of ADHD medication (Table 2).

**Table 2:** Psychiatric medications

ID	Medication (indication)
1	
2^a^	Melatonin (sleep)
3	Melatonin (sleep); clonidine (ADHD); quetiapine, amitriptyline, oxcarbazepine (irritability/aggression)
4^a^	
5	
6	
7	Trazodone and clonidine (sleep); risperidone and lorazepam (irritability/aggression)
8	Hydroxyzine (past) (anxiety, impulsivity)
9^a^	
10	Trazodone and melatonin (sleep); clonidine (ADHD); aripiprazole (irritability/aggression)
11	Dextroamphetamine as needed (ADHD)

Psychiatric medications and indications for each participant.

^a^Participant had a diagnosis of ADHD, confirmed in this study, but was not receiving treatment.

Eight of 11 participants were reported to present with anxiety. Two individuals presented with obsessive–compulsive disorder (OCD) symptoms—one had a prior diagnosis of OCD. Two presented with aggression and/or self-injury and one with pica. Four participants were receiving medication for mood and/or aggression, including aripiprazole, risperidone, lorazepam, quetiapine, and hydroxyzine.

On the Vineland-3, participants had similar levels of internalizing and externalizing behaviors (Table 1), scoring on average 20.4 (1.3) and 20.0 (1.3) in each subdomain respectively, about 1.5 standard deviations above the mean.

The Child Behavior Checklist (CBCL) total *T*-score ranged from 43 to 81 (63.7 ± 12.8), 7 of 11 participants’ scores surpass the clinical cutoff (*T* score > 65, per manual). Six individuals scored above the clinical threshold on the CBCL’s Depressive domain (63.8 ± 10.9), five in the ADHD domain (65.4 ± 9.1), and four in both the Anxiety (63.4 ± 10.9) and Oppositional/Defiant domains (59.2 ± 7.7). On the Aberrant Behavior Checklist (ABC), four individuals scored above the clinical threshold (*T* score > 60, per manual) in the Irritability subdomain, two in the Inappropriate Speech domain, and one in the Hyperactivity domain. Average *T* scores for the domains were 52.3 (9.6) in Irritability, 52.1 (10.1) in Inappropriate Speech, 50.5 (7.0) in Hyperactivity, 47.5 (7.1) in Lethargy/Social Withdrawal, and 46.9 (6.5) in Stereotypy.

The CBCL performed best at predicting ADHD status in our cohort, with five true positives, one false negative, and no false positives (Fig. 4). The ABC had the most false negatives and only assigned one true positive. This may be because the CBCL domain included items for hyperactivity, inattention, and impulsivity, while the ABC domain was focused on hyperactivity alone. There was one individual who had a DSM-5 diagnosis of ADHD but did not meet the clinical threshold on either of the measures; however, upon further inspection, the participant’s CBCL score was only two points away from the clinical threshold.

### Quality of life

Participants’ caregivers completed the Child and Family Quality of Life (CFQL-2). On average, caregivers rated the Family (3.89 ± 0.98), Caregiver (3.14 ± 0.90), and Child (2.93 ± 0.69) quality of life domains as most affected by their child’s diagnosis of CHAMP1 disorder. Caregivers were less affected in the Financial (2.91 ± 0.84), Relationship (2.86 ± 1.12), and Coping (2.85 ± 0.69) quality of life domains, and they reported that their Social Network quality of life (2.55 ± 0.97) was least affected. The highest rated negative and positive statements were ‘My child’s difficulties have added stress to our home life’ and ‘my child appears happy and content’ where average answers were *Agree* and *Often*, respectively.

On the CBCL, caregivers responded to both their greatest concerns about their children and the best things about their children. Six caregivers reported that language ability was one of their top concerns, six reported other DD (motor delays, toilet training, and self-help) as a top concern, and three reported behavioral abnormalities as their top concern. In response to what is best about their children, 10 caregivers described their child as both loving/affectionate and happy/joyful, and 5 caregivers reported that their children are friendly to everyone they meet.

### Medical comorbidities

#### Frequent (50%+)

The most common medical comorbidity was hypotonia, which was present in all 11 individuals (Supplementary Material, Table S4). Current hypotonia was most often assessed as mild by the neurologist, although five participants had a history of more severe hypotonia in early infancy, which often presented with feeding difficulties. Gait was directly assessed by a neurologist for 10 individuals; all had abnormal gait. Common findings were ataxic gait (*n* = 4) and hypotonic gait (*n* = 3). Hyperextensibility was present in 9 of 11 participants. Gastrointestinal abnormalities, present in 9 of 11 individuals, included constipation (8 of 11), gastrointestinal reflux (6 of 11), cyclical vomiting (6 of 11), and diarrhea (1 of 11). Eight participants were treated for these abnormalities: five for constipation, three for reflux, and three for cyclical vomiting.

Visual abnormalities were present in 8 of 11 participants, with specific findings of hyperopia (4 of 11), astigmatism (2 of 11), and amblyopia (2 of 11). Ocular abnormalities were also present in 8 of 11, with 5 of 11 having strabismus, 5 of 11 (four current) with nystagmus, and 1 of 11 with a unilateral coloboma.

Feeding issues were present in 8 of 11 individuals. Sleep disturbance was present in 7 of 11 and most often included difficulty staying asleep (5 of 11), followed by difficulty falling asleep (3 of 11), restless leg syndrome (1 of 11), and sleep apnea (1 of 11). Four participants were receiving treatment for sleep abnormalities, two receiving melatonin alone, one receiving melatonin and trazodone in combination, and one receiving trazodone and clonidine in combination. Dental abnormalities were also present in 7 of 11 participants, where the most common abnormality was retained primary teeth (4 of 7 individuals old enough for finding to be noticeable). Seven of 11 participants had allergies; 5 had seasonal allergies, and 1 of each had mold, food, or penicillin allergies. Six of 11 participants had head abnormalities: 5 of 11 with microcephaly, 2 of 11 with plagiocephaly, and 1 of 11 with macrocephaly.

#### Common (20–50%)

Recurrent infections, defined as two or more severe infections (requires antibiotics or hospitalization) in one year, three or more respiratory infections in one year, or the need for antibiotics for two months within one year, were present in 4 of 11 participants. Seizures were present in 4 of 11 participants, three with a history of a single febrile seizure and the other with a history of a single febrile seizure and a generalized seizure. One participant had a history of an abnormal electroencephalography, though had never had a seizure. Three of 11 participants required a neonatal intensive care unit (NICU) stay, and 1 of 11 was born prematurely. Additionally, three of nine participants who had undergone an MRI had prominent extra-axial spaces; the remaining seven participants who had an MRI had normal results.

#### Other less commonly reported features (<20%)

Hearing abnormalities were present in 2 of 11 participants, both with bilateral loss. Endocrinology abnormalities were present in 2 of 11 individuals, one with hypothyroidism and one with diabetes mellitus. Features displayed by only one participant include migraines, vesicoureteral reflux, immunodeficiency (as reflected by low T cell counts), and neutropenia.

### Dysmorphology

The most common dysmorphic feature was a wide nasal bridge, present in 10 of 11 participants. Eight had a bulbous nose, seven had hypoplastic nails, and six had ear anomalies, which included three with low set ears, two with protruding ears, one with large ears, and one with a preauricular sinus dimple. Five participants had a pointed chin, five had epicanthal folds, four had fleshy hands, four had fifth finger clinodactyly, three had hypertelorism, three had a flat midface, three had a high arched palate, three had two-three toe syndactyly, two had full lips, and two had a short neck. Features present in 1 of 11 include micrognathia, full cheeks, malar hypoplasia, periorbital fullness, deep-set eyes, long eyelashes, and sparse hair. Photos of participants are in Figure 1.

## Discussion

This study represents the first prospective comprehensive evaluation of individuals with CHAMP1 disorder. The assessments included multiday clinician-administered evaluations by an interdisciplinary team of clinical researchers. Psychiatric, behavioral, ASD, language, and sensory features were specifically evaluated to fill a gap in the current literature, which has previously focused on medical and dysmorphic features (Table 3).

**Table 3:** Results from our cohort and previous literature

	Current study	Hempel, 2015	Tanaka, 2016	Isidor, 2016	Okamoto, 2017	Garrity, 2021	All	%
Sample size (previously reported)	11 (3)	5 (0)	5 (0)	6 (0)	1 (0)	14 (2)	38	
Mean age (SD)	10.7 (7.8)	7.2 (6.4)	10.4 (7.6)	7.8 (2.2)	6.3	9.3 (6.8)		
Female, male	8, 3	2, 3	5, 0	3, 3	0, 1	8, 6	23, 15	
DD	11/11	5/5	5/5	6/6	1/1	14/14	38/38	100
Motor delay	11/11	5/5	5/5	6/6	1/1	9/9	38/38	100
Speech delay	11/11	5/5	5/5	6/6	1/1	9/9	38/38	100
ID	11/11	5/5	5/5	6/6	1/1	14/14	38/38	100
Hypotonia	11/11	5/5	4/5	5/5	1/1	14/14	37/38	97
Gait abnormalities	10/11	3/5	n/d	2/2	n/d	n/d	14/17	82
Dental abnormalities	7/11	4/5	n/d	n/d	n/d	n/d	11/15	73
Anxiety	8/11	n/d	n/d	n/d	n/d	n/d	8/11	73
Verbal	8/11	3/5	2/5	3/6	1/1	7/10	22/33	67
Ocular abnormalities	8/11	2/5	4/5	3/5	0/1	8/14	25/38	66
GI abnormalities	9/11	2/5	2/5	2/6	n/d	11/14	21/32	66
Allergies	7/11	n/d	n/d	n/d	n/d	n/d	7/11	64
Microcephaly	5/11	3/5	4/5	3/6	1/1	8/14	23/38	61
Visual abnormalities	8/11	3/5	0/5	5/5	1/1	8/14	22/38	58
ADHD	6/10	n/d	2/5	n/d	n/d	n/d	8/15	53
Abnormal MRI brain	3/9	2/5	3/4	0/5	1/1	2/3	14/27	52
Sleep problems	7/11	3/5	4/5	2/6	n/d	5/14	18/37	49
Recurrent infections	4/11	3/5	n/d	n/d	n/d	7/10 Unknown on recurrent	7/16	44
ASD	3/9	1/5	n/d	1/6	n/d	5/13 (ASD or features)	9/30	30
NICU stay	3/11	n/d	n/d	n/d	n/d	n/d	3/11	27
Seizures	4/11	1/5	2/5	1/4	1/1	9/14	10/38	26
Hearing abnormalities	2/11	n/d	3/5	n/d	n/d	1/13	5/27	19
Endocrine abnormalities	2/11	n/d	n/d	n/d	n/d	n/d	2/11	18
Renal/urinary tract abnormalities	1/11	1/5	n/d	0/5	n/d	n/d	2/21	10
Preterm birth	1/11	0/5	1/5	0/6	0/1	n/d	2/27	7

Comorbidities reported in previous literature, in descending order of prevalence. Wang *et al*. (2020) was excluded, as the participant from the case report was only 6 months old and the features here were therefore mostly not applicable (28). Abbreviations: ADHD: Attention-deficit/hyperactivity disorder; ASD: Autism spectrum disorder; DD: Developmental delay; ID: Intellectual disability; GI: gastrointestinal; NICU: Neonatal Intensive Care Unit; n/d: Not done

ASD symptoms were directly evaluated for the first time in this study, whereas previous literature reported community diagnoses (1/5, Hempel *et al*. 2015) or ASD traits (5/13, Garrity *et al*., 2021) without noting standardized assessment. Consensus DSM-5 diagnoses of ASD were established for three of nine individuals (33%) based on the psychiatric evaluation, the CARS-2^obs^, and the ADI-R. Results on the ADI-R in this cohort had a high false positive rate (33%), a phenomenon often seen in complex neurodevelopmental conditions due to comorbid ID. However, the sample size is limited. The CARS-2^obs^, which was used as a remote substitution of the Autism Diagnostic Observation Schedule (ADOS-2), performed marginally better with two false positives. All individuals with a consensus diagnosis of ASD met on both the CARS-2^obs^ and the ADI-R. There was one individual who met on both assessments but did not have ASD—this participant showed clear social strengths, including well-modulated eye contact, initiating interactions, and engaging in social games, and had a range of facial expressions. However, this participant was difficult to engage in more structured remote assessment, which likely attributed to the false positive on the CARS-2^obs^. Additionally, regardless of DSM-5 ASD diagnosis, many individuals in our cohort presented with features of ASD. Most individuals, regardless of ASD status, presented with RRBs and sensory symptoms, a finding seen in other genetic NDDs (26,27). Both the SAND and the Sensory Experiences Questionnaire Version 3.0 (SEQ-3.0) indicated that individuals had high levels of sensory-seeking behaviors. While only three participants met full DSM-5 criteria for ASD, all participants in our cohort demonstrated some ASD traits, and Vineland-3 Adaptive Behavior Composite scores indicate all likely present with ID. Importantly, many individuals with neurogenetic syndromes associated with ASD and ID would benefit from evidence-based autism therapies, such as Applied Behavior Analysis.

Additional psychiatric findings from our study included a high rate of ADHD (60%) and OCD traits (18%). Half of those diagnosed with ADHD were receiving pharmacological interventions. We found that the CBCL performed best at assessing ADHD features in our cohort, as compared to the ABC Hyperactivity domain. There were also high rates of anxiety reported in this cohort (73%), with three participants having separation anxiety. Despite this, in line with previous reports, many individuals presented with a happy and joyful demeanor (19). This was observed directly by clinicians as well as reported from caregivers and represents an area of strength.

We observed wide variability in cognitive and adaptive functioning, ranging from severely impaired to scores within the low average range. One explanation for the variability is the wide age range within our cohort (1.6–28.3 years). Domain scores on the DP-4 and Vineland-3 were significantly negatively associated with age, an expected finding. The youngest participant in our cohort had the highest scores in all domains on both assessments, and the oldest participant had the lowest scores. Because expectations for developmental and adaptive functioning skills are lower in younger children, standard scores—which compare current skills to those expected for age—tend to be higher. As the breadth of skills expected increases with age, the margin of deficits in these individuals becomes wider, and scores therefore commonly decrease with age. In addition to age, we found that microcephaly was associated with lower scores on the Vineland-3 Adaptive Behavior Composite, Communication, and Daily Living Skills domains.

Previous literature has noted differing rates of verbal ability; combining all studies to date, 67% of assessed individuals with CHAMP1 disorder attained verbal language, at minimum at the single-word level. Results from clinician-administered direct assessments, caregiver interviews, and caregiver questionnaires indicated that most individuals in our cohort had higher receptive vocabulary and language ability compared to expressive. Most of our cohort utilized single words (9 of 11) while only four used spontaneous phrase speech. However, three participants were still younger than this cohort’s average age of phrase speech acquisition at the time of assessment and may achieve this milestone later on. There was no report of language regression.

Medical features are the most thoroughly described comorbidities in previous literature. Our study found similar rates of common features such as microcephaly, hypotonia, visual abnormalities, dysmorphic features, and gastrointestinal abnormalities. Gait abnormalities were prospectively assessed for the first time and found to be universally present in this cohort (10 of 10). Additionally, endocrine abnormalities (hypothyroidism, diabetes mellitus) were present in 2 of 11 participants, a new finding to the literature. Interestingly, four of seven individuals had retained primary teeth. In another NDD, ADNP syndrome, the majority of individuals have premature primary tooth eruption (29). *ADNP* and *CHAMP1* interact with similar genes and pathways, including *HP1.* While not the same abnormalities, given the biological connection between the genes, it is interesting that both conditions have primary tooth abnormalities and may hint at shared pathways.

The battery of assessments utilized in this study was designed to be conducted fully remotely due to safety concerns of in-person testing during the COVID-19 pandemic. However, beyond the specific need for a virtual protocol, we found concrete benefits of conducting the study remotely. First, clinicians were able to observe and assess participants in their homes. Typically, families travel to our center for multiday evaluations, and the change in schedule and environment can impact behavior. Assessing individuals in their natural environments may mitigate these difficulties and lead to more accurate assessments of behavior that are representative of everyday life. Second, while socioeconomic status and geographic location are typically barriers to research participation, we were able to increase accessibility in this study. The remote battery eliminated the need for families to take off multiple days of work and the financial burden of having to travel to complete the study.

However, there were also limitations to the remote battery, including the inability to complete both traditional cognitive testing and the ADOS-2. While ASD consensus diagnoses were given with high certainty in most cases, we deferred one diagnosis because of insufficient evidence with the available format. The addition of the ADOS-2 could have helped to clarify ASD status in this participant. Additionally, there was one participant who did not complete the full battery because of loss to follow-up, which may have been prevented with in-person visits. Overall, we found the benefits of the remote study outweighed the limitations. In the future, we will likely utilize a combination of remote and in-person assessments to reduce the burden to families while also comprehensively collecting data. Co-norming this battery of assessments is also an important future direction and is necessary to optimally examine which domains are most versus least impaired. Co-norming will offer a useful metric at both the group and individual level, particularly in syndromes such as CHAMP1 that often result in impairment across multiple domains of functioning.

Overall, this study provides a comprehensive neurobehavioral profile of individuals with CHAMP1 disorder. While rates of ASD are relatively low in this rare disorder compared to others such as tuberous sclerosis or ADNP syndrome, they are higher than some other disorders already included on clinical autism sequencing panels (e.g. FOXP1). Though the sample size was small, our results provide evidence that ASD is a common comorbidity in CHAMP1 disorder, and *CHAMP1* therefore warrants inclusion on ASD-focused sequencing panels. Sensory reactivity symptoms were also notably common and suggest the importance of thorough assessments of the sensory domain and the potential utility of related interventions such as occupational therapy. Future studies can also investigate whether sensory symptoms are affecting other core domains such as social and behavioral features. High rates of ADHD (60%) but low rates of consistent treatment (50% of those with ADHD) indicate that treatment for ADHD is underutilized and provides another promising target of therapeutic intervention for individuals with CHAMP1 disorder. Additionally, a review of clinical measure performance in this cohort may aid in clinical trial design to determine optimal endpoints and clinical outcome assessments. Importantly, this study provides further characterization of sensory, language, motor and medical features and serves as a basis for the feasibility of future remote phenotyping studies in rare neurogenetic syndrome.

## Materials and Methods

### Participants

Participants ranged from 1.6 to 28.3 years of age (10.7 ± 7.8) and included eight females and three males. All participants had a diagnosis of CHAMP1-related NDD, confirmed by a likely pathogenic or pathogenic sequence variant in the *CHAMP1* gene as classified by the American College of Medical Genetics and Genomics and Association for Molecular Pathology guidelines (30).

Clinical assessments included approximately four to six hours of direct evaluation of the participant, six hours of caregiver interviews, and four hours of caregiver questionnaires (Fig. 6). All direct evaluations and caregiver interviews were conducted remotely using videoconferencing (HIPAA-compliant Zoom). Questionnaires were administered virtually using an online system, REDCap. Assessments were administered by a child and adolescent psychiatrist, clinical psychologists, a pediatric neurologist, and a genetic counselor. Additionally, medical records were reviewed by the study psychiatrist to supplement medical history information.

**Table 4:** Phenotyping battery.

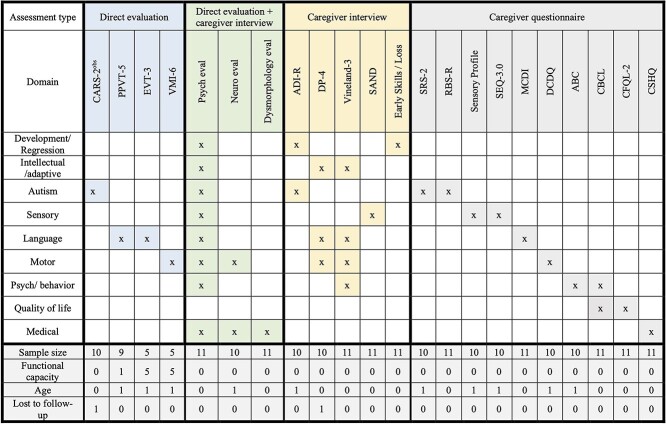

Phenotyping battery. Summary of clinical battery; all assessments were completed remotely. Crosses indicate which domains the data from each assessment contributed to. The ‘Sample size’ row represents the sample size of each assessment; when not all 11 participants completed an assessment, the bottom three rows indicate the reason: (a) the participant did not have the functional capacity to complete the assessment, (b) the participant was not within the age range of the assessment, or (c) the participant was lost to follow-up. *Abbreviations*: ABC: Aberrant Behavior Checklist; ADI-R: Autism Diagnostic Interview-Revised; ASD: Autism spectrum disorder; CARS-2^obs^: Childhood Autism Rating Scale; CBCL: Child Behavior Checklist; CFQL-2: Child and Family Quality of Life-Second Edition; CSHQ: Child Sleep Habits Questionnaire; DCDQ: Developmental Coordination Disorder Questionnaire; DP-4: Developmental Profile 4; EVT-3: Expressive Vocabulary Test Third Edition; MCDI: MacArthur Bates Communicative Indices; PPVT-5: Peabody Picture Vocabulary Test Fifth Edition; RBS-R: Repetitive Behavior Scale-Revised; SAND: Sensory Assessment for Neurodevelopmental Disorders; SEQ-3.0: Sensory Experiences Questionnaire Version 3.0; SRS-2: Social Responsiveness Scale, Second Edition; Vineland-3: Vineland Adaptive Behavior Scales, Third Edition; VMI-6: Visual Motor Integration, Sixth Edition.

### Development and regression

Developmental milestones were collected using the ADI-R (31), the Early Skills Attainment and Loss (32), and by caregiver interview. Skill loss was assessed by the Early Skills Attainment and Loss (32).

### Cognitive and adaptive functioning

The Developmental Profile 4 (33) was used to approximate cognitive functioning, as traditional cognitive assessments require in-person testing and were not feasible due to the COVID-19 pandemic. Standard scores are normed up to 21 years; the participant in the cohort over this age was scored using the 21-year norms, as her scores were already at the floor. Adaptive functioning was assessed by the Vineland-3 Survey Interview Form (34). Consensus diagnoses of DSM-5 (35) ID were ascertained by the study clinicians based on results from these two assessments.

### Autism symptomatology

The CARS-2^obs^ (36) was used as a direct assessment of ASD features, in addition to the ADI-R and psychiatric evaluation. Consensus diagnoses were determined after a discussion of the results from these assessments with the study clinicians. Reliability was established for the clinicians administering the CARS-2^obs^ prior to study onset, based on assessments with both typically developing children and those with NDDs. In addition, caregiver questionnaires were used to further describe ASD features: the SRS-2 (37) and the RBS-R (38).

The SAND is a direct observational assessment and corresponding caregiver interview (39). Because assessments were remote, only the caregiver interview portion was completed. Caregivers also completed the SP (40) and the SEQ-3.0 (41), which further described sensory symptoms.

### Language and communication

The PPVT-5 (42) directly assessed receptive vocabulary, and the EVT-3 (43) directly assessed expressive vocabulary. Those who could not complete the PPVT-5 and/or EVT-3 due to functional capacity were given a raw score of 0 and a standard score of 40 (floor). The Vineland-3 measured communication, with subdomains of Expressive, Receptive, and Written communication. The Vineland-3 subdomains are measured in V-scale scores, which have a mean of 15 and a standard deviation of 3. The DP-4 Communication domain was also administered. Lastly, the MCDI was used to quantify words understood and spoken (44).

### Motor

The VMI-6 (45) assessed fine motor and visual integration skills. The VMI-6 protocol was mailed to families ahead of time and administered by a clinician based on guidelines described by Pearson through videoconferencing. For those with a raw score at the floor (standard score of 45), a score of 42 was given. Developmental coordination disorders were screened for using the DCDQ (46). The Vineland-3 Motor domain was administered to individuals 7 years and younger. Additionally, the DP-4 Physical domain was used to assess motor skills.

### Psychiatric and behavioral comorbidities

Psychiatric comorbidities were evaluated during the psychiatric evaluation and using the ABC (47) and the CBCL. ABC *T*-scores were calculated based on age (*n* = 10) or by intellectual quotient under 70 (*n* = 1), per the manual (48). Consensus DSM-5 diagnoses of ADHD were ascertained after a discussion among the study psychiatrist and psychologists as well as a review of past records.

#### Quality of life

The CFQL-2 (49) was used to assess the quality of life.

#### Medical comorbidities

Medical comorbidities were assessed during the psychiatric evaluation and supplemented with the review of prior medical records by the study psychiatrist. The neurological evaluation assessed gait, tone and motor coordination through remote assessment and by review of videos collected for this purpose. Dysmorphisms were assessed by a genetic counselor through videoconferencing and caregiver interview.

## Supplementary Material

Supplementary_Tables_1-4_ddac018
